# Prophylactic drug management for febrile seizures in children

**DOI:** 10.1590/1516-3180.20131314T1

**Published:** 2013-08-01

**Authors:** Martin Offringa, Richard Newton

## Abstract

**BACKGROUND:**

Febrile seizures occurring in a child older than one month during an episode of fever affect 2% to 4% of children in Great Britain and the United States and recur in 30%. Rapid-acting antiepileptics and antipyretics given during subsequent fever episodes have been used to avoid the adverse effects of continuous antiepileptic drugs.

**OBJECTIVE:**

To evaluate the effectiveness and safety of antiepileptic and antipyretic drugs used prophylactically to treat children with febrile seizures.

**METHODS:**

*Search methods:* We searched the Cochrane Central Register of Controlled Trials (CENTRAL) (The Cochrane Library 2011. Issue 3); MEDLINE (1966 to May 2011); EMBASE (1966 to May 2011); Database of Abstracts of Reviews of Effectiveness (DARE) (May 2011). No language restrictions were imposed. We also contacted researchers in the field to identify continuing or unpublished studies.

*Selection criteria:* Trials using randomized or quasi-randomized patient allocation that compared the use of antiepileptic or antipyretic agents with each other, placebo or no treatment.

*Data collection and analysis:* Two review authors (RN and MO) independently applied pre-defined criteria to select trials for inclusion and extracted the pre-defined relevant data, recording methods for randomization, blinding and exclusions. Outcomes assessed were seizure recurrence at 6, 12, 18, 24, 36 months and at age 5 to 6 years in the intervention and non-intervention groups, and adverse medication effects. The presence of publication bias was assessed using funnel plots.

**MAIN RESULTS:**

Thirty-six articles describing 26 randomized trials with 2740 randomized participants were included. Thirteen interventions of continuous or intermittent prophylaxis and their control treatments were analyzed. Methodological quality was moderate to poor in most studies. We could not do a meta-analysis for 8 of the 13 comparisons due to insufficient numbers of trials. No significant benefit for valproate, pyridoxine, intermittent phenobarbitone or ibuprofen versus placebo or no treatment was found; nor for diclofenac versus placebo followed by ibuprofen, acetominophen or placebo; nor for intermittent rectal diazepam versus intermittent valproate, nor phenobarbitone versus intermittent rectal diazepam.

**AUTHORS’ CONCLUSIONS:**

No clinically important benefits for children with febrile seizures were found for intermittent oral diazepam, phenytoin, phenobarbitone, intermittent rectal diazepam, valproate, pyridoxine, intermittent phenobarbitone or intermittent ibuprofen, nor for diclofenac versus placebo followed by ibuprofen, acetominophen or placebo. Adverse effects were reported in up to 30% of children. Apparent benefit for clobazam treatment in one recent trial needs to be replicated to be judged reliable. Given the benign nature of recurrent febrile seizures, and the high prevalence of adverse effects of these drugs, parents and families should be supported with adequate contact details of medical services and information on recurrence, first aid management and, most importantly, the benign nature of the phenomenon.

**This is the abstract of a Cochrane Review published in the Cochrane Database of Systematic Reviews (CDSR) 2013, issue 4, Art. No. CD003031. DOI:** 10.1002/14651858.CD003031.pub4 (http://onlinelibrary.wiley.com/doi/10.1002/14651858.CD003031.pub2/abstract). For full citation and authors details, see reference 1.

**The full text is freely available from:** http://cochrane.bireme.br/cochrane/main.php?lang=pt&lib=COC (this link may be temporary)
